# Vessel Fractional Flow Reserve and Graft Vasculopathy in Heart Transplant Recipients

**DOI:** 10.1155/2020/9835151

**Published:** 2020-07-12

**Authors:** Sakura Nagumo, Emanuele Gallinoro, Alessandro Candreva, Takuya Mizukami, Giovanni Monizzi, Monika Kodeboina, Sofie Verstreken, Riet Dierckx, Ward Heggermont, Jozef Bartunek, Marc Goethals, Dimitri Buytaert, Bernard De Bruyne, Jeroen Sonck, Carlos Collet, Marc Vanderheyden

**Affiliations:** ^1^Cardiovascular Center Aalst, OLV Clinic, 9300 Aalst, Belgium; ^2^Division of Cardiology, Department of Internal Medicine, Showa University Fujigaoka Hospital, Yokohama, 2278501 Kanagawa, Japan; ^3^Department of Translational Medical Sciences, University of Campania “Luigi Vanvitelli”, 80131 Naples, Italy; ^4^Department of Advanced Biomedical Sciences, Federico II University, 80131 Naples, Italy; ^5^Cardiovascular Research Center Maastricht, Maastricht, AZ-6202, Netherlands; ^6^Department of Cardiology, Lausanne University Hospital, 1011 Lausanne, Switzerland

## Abstract

**Background:**

Cardiac allograft vasculopathy (CAV) remains the Achilles' heel of long-term survival after heart transplantation (HTx). The severity and extent of CAV is graded with conventional coronary angiography (COR) which has several limitations. Recently, vessel fractional flow reserve (vFFR) derived from COR has emerged as a diagnostic computational tool to quantify the functional severity of coronary artery disease.

**Purpose:**

The present study assessed the usefulness of vFFR to detect CAV in HTx recipients.

**Methods:**

In HTx patients referred for annual check-up, undergoing surveillance COR, the extent of CAV was graded according to the criteria proposed by the international society of heart and lung transplantation (ISHLT). In addition, three-dimensional coronary geometries were constructed from COR to calculate pressure losses using vFFR.

**Results:**

In 65 HTx patients with a mean age of 53.7 ± 10.1 years, 8.5 years (IQR 1.90, 15.2) years after HTx, a total number of 173 vessels (59 LAD, 61 LCX, and 53 RCA) were analyzed. The mean vFFR was 0.84 ± 0.15 and median was 0.88 (IQR 0.79, 0.94). A vFFR ≤ 0.80 was present in 24 patients (48 vessels). HTx patients with a history of ischemic cardiomyopathy (ICMP) had numerically lower vFFR as compared to those with non-ICMP (0.70 ± 0.22 vs. 0.79 ± 0.13, *p* = 0.06). The use of vFFR reclassified 31.9% of patients compared to the anatomical ISHLT criteria. Despite a CAV score of 0, a pathological vFFR ≤ 0.80 was detected in 8 patients (34.8%).

**Conclusion:**

The impairment in epicardial conductance assessed by vFFR in a subgroup of patients without CAV according to standard ISHLT criteria suggests the presence of a diffuse vasculopathy undetectable by conventional angiography. Therefore, we speculate that vFFR may be useful in risk stratification after HTx.

## 1. Introduction

Cardiac transplant-related arteriopathy remains a leading cause of morbidity and mortality in heart transplant recipients with one in 3 patients developing cardiac allograft vasculopathy (CAV) in the first 5 years after heart transplantation [1]. In contrast to the focal, proximal epicardial lesions in atherosclerosis, CAV affects both epicardial and intramural vessels and is characterized by progressive intimal proliferation in its early stage and by luminal narrowing and microvascular dysfunction in its later stage [2, 3]. Its etiology is likely complex and is thought to involve an interplay between immunologic (human leukocyte antigen and other mismatches), infectious (cytomegalovirus and others), and classical atherosclerotic risk factors (lipid status, diabetes, and others).

Nowadays, a grading system based on coronary angiography is recommended by the international society of heart and lung transplantation (ISHLT) to evaluate the severity and extent of CAV [4]. However, coronary angiography lacks the resolution to diagnose early as well as diffuse stages of CAV, a limitation that has partly been overcome by more recent imaging techniques, such as intravascular ultrasound (IVUS) [5, 6]. On his turn, however, IVUS findings are unable to assess the functional significance of vascular disease. In this regard, fractional flow reserve (FFR), an index to identify epicardial disease responsible for myocardial ischemia might be helpful. In heart transplant patients, an abnormal epicardial physiology on the basis of an FFR <0.90 predicts worse clinical outcome, defined by the cumulative survival free of death or retransplantation [7].

While FFR can be measured during routine coronary angiography using a pressure sensor guidewire to calculate the ratio between coronary pressure distal to a coronary artery stenosis and aortic pressure under conditions of hyperemia, FFR can also be computed from the 3-dimensional reconstruction of the coronary artery obtained from invasive coronary angiography using computational fluid dynamics calculations or by a mathematical approach. Angiography-based FFR (vFFR) estimates have been shown to perform well against invasive FFR and have a high diagnostic performance against dichotomous FFR categorization. Angiography-based FFR (vFFR) estimates have been shown to perform well against invasive FFR and have a high diagnostic performance against dichotomous FFR categorization [8, 9]. However, data on the use of vFFR in heart transplant recipients are lacking.

Therefore, the aim of the present study was to evaluate CAV by comparing the standard ISHLT grading system with functional vFFR measurements.

## 2. Methods

### 2.1. Study Design

This retrospective study was performed in all patients who underwent HTx at the Cardiovascular Center Aalst between January 1987 and December 2018. Heart transplant recipients were included at time of their yearly annual surveillance coronary angiography, and patients with angiograms amenable for three-dimensional vessel reconstruction were included in the study. In these patients, the angiography-derived FFR was derived from the latest angiogram. In those patients where serial angiographies were available, the vFFR was calculated to evaluate the functional progression of CAV. All patients provided written informed consent according to local institutional practice.

### 2.2. Cardiac Allograft Vasculopathy Classification

The presence and extent of cardiac allograft vasculopathy was graded according to the standard ISHLT criteria as previously described [4]. Briefly, CAV was classified as absent (CAV 0), mild (CAV 1), moderate (CAV 2), or severe (CAV 3) according to the ISHLT classification. Patients with any significant lesions were classified as CAV 3 if an echocardiogram or left ventricular angiogram, performed anytime within 6 months of angiography, reported a left ventricular ejection fraction of ≤45%. All the analyses were performed by an independent core laboratory.

### 2.3. Angiography-Derived FFR

The angiography-derived FFR was performed using vessel FFR software (vFFR, CAAS 8.2 Software, Pie Medical Imaging, Maastricht, the Netherlands). For the calculation of vFFR, 2 projections of at least 30 degrees of difference in angulation/rotation are used to create a 3D reconstruction of the coronary artery. Temporal alignment of the cardiac cycle was performed automatically by electrocardiogram (ECG) triggering. Contour detection was automatic, and manual correction was allowed and recorded. The pressure drop was calculated by applying physical loss of blood behavior with patient-specific aortic pressure. vFFR was calculated as the ratio of distal coronary pressure to aortic pressure. vFFR values were obtained for each major native coronary vessel. The most distal value was used for the analysis, and vFFR values ≤0.80 were considered as significant disease.

In cases of the serial vFFR analysis, angiographies acquired in projections with 30 degrees of the previous angiographies were utilized. vFFR values were visually matched using anatomical landmarks. For the patient-level analysis, the lowest vFFR value of the 3 major epicardial vessels was selected. Significant functional progression was defined as change in vFFR higher than two standard deviation of the interobserver reproducibility [9]. All the analyses were performed by an independent core laboratory.

### 2.4. Clinical Follow-Up

Adverse events were collected through the hospital database system. Death was defined as death from any cause. Spontaneous myocardial infarction was defined according to the 4^th^ universal definition [10]. Target vessel revascularization was defined as either percutaneous or surgical revascularization. Medical therapy with immunosuppressive agents was prescribed according to the hospital protocol.

### 2.5. Statistical Analysis

Continuous variables are expressed as mean ± standard deviation, and categorical variables are expressed as count and percentages. Nonnormally distributed variables are expressed as median and interquartile range (IQR). Continuous variables were compared using the *t*-test, Mann–Whitney *U* test, Wilcoxon matched-paired test, or Kruskal–Wallis test according to the variable distribution. Categorical variables were compared with the chi-square or Fisher's test, as appropriate. Agreement between the ISHLT CAV grade and the vFFR value was investigated using weighted Cohen's kappa. All Statistical analyses were performed using R software (version 3.5.3). *p* values <0.05 were considered statistically significant.

## 3. Results

### 3.1. Baseline Characteristics

**Figure 1:**
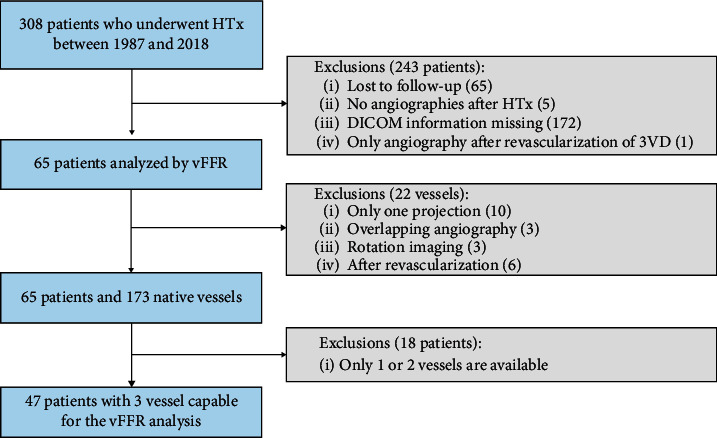
A total number of 65 patients who underwent heart transplant and in whom one or more vessels could be analyzed using vFFR were included. Subsequently, the vFFR value of all three native coronary arteries and the ISHLT CAV grade were compared. HTx, heart transplantation; vFFR, vessel fractional flow reserve; ISHLT, international society of heart and lung transplantation; and CAV, cardiac allograft vasculopathy.

Figure 1 depicts the flow diagram illustrating the selection of the study population. In 65 patients who underwent heart transplantation between April 1987 and December 2018, one or more vessels could be analyzed using vFFR. In 47 of them, all three vessels could be analyzed. The clinical characteristics are summarized in Table 1. The median time between heart transplantation and last coronary angiography was 8.5 years (IQR 1.90, 15.2). Most donors (76.9%) and recipients (67.7%) were male. The mean donor and the mean recipient ages were 35.7 and 53.7 years, respectively. The most frequent indication for HTx was ischemic cardiomyopathy. All patients were treated with calcineurin inhibitors and 75.4% of patients with an antimetabolite (mycophenolate mofetil in 72.3% and azathioprine in 3.1%).

### 3.2. Functional and Angiographic Classification

Overall, 173 native vessels in 65 patients were analyzed (59 (34.1%) LAD, 61 (35.3%) LCX, and 53 (30.6%) RCA). Among 47 patients in whom the three-vessel FFR analysis was feasible, ISHLT CAV grading was assessed. Based on the grading, 23 patients were categorized as CAV 0, 12 patients as CAV 1, 4 patients as CAV 2, and 8 patients as CAV 3.

The functional and anatomical vessel characteristics are summarized in Table 2. The minimal lumen diameter was 1.70 ± 0.69 mm with a reference vessel diameter of 2.60 ± 0.84 mm and percent diameter stenosis was 35.1 ± 14.0%. The mean vFFR in the study population was 0.84 ± 0.15 with 27.7% of vessels having a vFFR ≤ 0.80. Patients with a history of ischemic cardiomyopathy (ICMP) had a trend towards lower vFFR values as compared to non-ICMP etiology (0.70 ± 0.22 vs. 0.79 ± 0.13, *p* = 0.06).

As expected, when categorizing functional vessel characteristics by CAV classification (Table 3), a significant lower vFFR (*p*=0.009) and a higher percent diameter stenosis (*p* < 0.001) were observed in patients with the higher CAV grade.

### 3.3. Agreement between ISHLT Classification of CAV and vFFR

**Figure 2:**
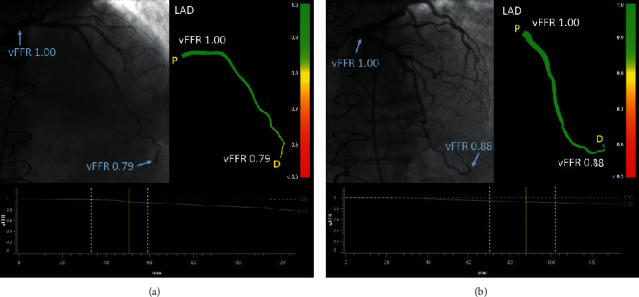
(a) Normal coronary angiogram with functionally significant CAV according to the vFFR analysis. The LAD shows no stenosis on angiography; however, the vFFR shows diffuse pressure losses, and the distal vFFR value is 0.79. (b) Normal angiogram without functionally significant CAV according to the vFFR analysis. The LAD shows no stenosis on angiography, and the vFFR shows small pressure losses resulting in a distal FFR value of 0.88. CAV, cardiac allograft vasculopathy; LAD, left anterior descending coronary artery; and vFFR, vessel fractional flow reserve.

Table 4 depicts the anatomical and functional evaluation stratified by the CAV grade and vFFR. When using a cut-off of 0.80, 34.8% of the patients were reclassified from the anatomically low-risk group (i.e. CAV 0) to functionally significant CAV (i.e. vFFR ≤ 0.80) (Table 4 and case examples in Figure 2). Overall, 31.9% of patients were reclassified by vFFR. The agreement between the anatomical CAV classification and the vFFR was fair (kappa = 0.34, 95% CI 0.08 to 0.65).

### 3.4. Progression of CAV

In 75 vessels (35 patients), serial coronary angiographies were available. The time difference between angiographic evaluations was 2.28 years (IQR 1.47, 3.91). Delta vFFR values decreased by 0.05 ± 0.10 units (*p* < 0.001). There was no statistical difference in the delta vFFR between patients with a history of ICMP or non-ICMP (0.17 (IQR 0.06, 0.24) vs. 0.07 (IQR 0.06, 0.11), *p* = 0.19). Thirty-seven vessels (22 patients) showed a significant functional progression over time.

### 3.5. Clinical Outcomes

During a median clinical follow-up of 10.9 years (IQR 4.21, 17.3), 14 patients (21.5%) experienced a major adverse cardiac event rate: 4 patients died (8.5%), 2 patients had acute myocardial infarction, and 10 patients underwent revascularization (8 PCI and 2 coronary artery bypass graft surgery).

## 4. Discussion

The main findings of the present study can be summarized as follows. The functional assessment of the coronary circulation using the angiography-derived FFR is feasible in heart transplant recipients and allowed to identify in 32% of coronary cases a significant functional impairment undetectable by the standard anatomical analysis according to ISHLT criteria. Second, using the serial analysis, the functional progression of CAV was captured by the angiography-derived FFR. Therefore, we speculate that vFFR may be a helpful tool in the evaluation of CAV and risk stratification post HTx.

### 4.1. Anatomical vs Functional Evaluation of CAV

CAV, a progressive and diffuse process involving both the epicardial coronary arteries and the microcirculation remains the major cause of late mortality in heart transplant recipients. Unlike native atherosclerotic disease, it develops more rapidly and is predominantly characterized by a diffuse intimal proliferation [11]. Although the ISHLT classification is a standard method of assessing CAV severity and has been described as an accurate predictor of long-term outcomes after heart transplant [12], the visual angiographic evaluation exhibits high variability particularly in CAV where the disease is diffuse [13]. Apart from these limitations, it has been well described that in atherosclerotic disease, there might be a significant discrepancy between the anatomical and physiological assessment of the lesion severity in up to 37% of cases [14]. Thus, the functional assessment of coronary lesion severity by invasive pressure-wire derived metrics such as fractional flow reserve (FFR) has been introduced in the surveillance and evaluation of not only atherosclerotic but also CAV coronary lesions [15, 16]. It was shown that fractional flow reserve (FFR) predicts adverse outcomes in patients with ischemic cardiomyopathy as well as in those who underwent heart transplantation. In CAV, FFR correlates with IVUS-assessed plaque volume and is abnormal in a significant proportion of asymptomatic cardiac transplant patients with normal angiograms [17, 18]. An abnormal FFR, soon after orthotopic heart transplantation (OHT), has been shown to be an independent predictor of long-term mortality or need for redo OHT [19]. These observations suggest a clear clinical role for the functional assessment of the coronary circulation after OHT.

### 4.2. Angiography-Derived FFR and CAV Grade

Recently, new technologies have been developed to estimate FFR from coronary angiography without using a coronary pressure wire [20]. The addition of blood flow simulation to 3D quantitative coronary angiography (QCA) has inaugurated a new era for the coronary angiography-based functional coronary hemodynamic assessment [21]. Compared to the invasive wire-derived FFR and regardless of computational approach and software packages, angiography-derived FFR has been shown highly accurate to detect hemodynamically significant lesion [8].

In the present study, angiography-derived FFR suggested the presence of functional disease in heart transplant recipients with coronary vessels considered angiographically “normal.” In 32% of coronary cases, a significant functional impairment undetectable by the standard anatomical analysis was observed. The use of vFFR reclassified 8 patients (34.8%) from an anatomically low-risk group (CAV 0) to a functionally high-risk group (Table 4). These observations are in line with the data of Fearon who also demonstrated in a substantial number of HTx patients an abnormal pressure wire-derived FFR despite angiographically normal coronary angiograms [18].

As HTx recipients have denervated hearts, they rarely present chest pain. Therefore, most transplant centers perform periodic coronary angiography for routine CAV and organ rejection surveillance [22]. Nonetheless, coronary angiography underestimates CAV because of the challenges identifying truly normal segments in cases of diffuse luminal reduction. Novel QCA software deriving FFR from a combination of quantitative angiographic parameters may help detecting CAV.

### 4.3. Clinical Implications

Coronary angiography remains the gold standard for detecting clinically overt CAV. The ISHLT CAV scoring bears prognostic information with worse prognosis in those HTx patients with CAV greater or equal to 2. Similarly, functional evaluation by FFR has also been shown to bear prognostic information. The introduction of vFFR might be an additional tool in the risk stratification of HTx patients since it not only helps to detect CAV earlier but also appears to be more sensitive. The most likely explanation is that the diffuse pattern of involvement in HTx patients might be overseen by standard coronary angiography. By adding the vFFR analysis in the grading of CAV, information about the extent of the disease is provided, which might be helpful in fine-tuning the appropriate immunosuppressive regimen. Further prospective studies that explore the role of vFFR in grading CAV severity are warranted.

### 4.4. Limitations

First, we only evaluated CAV by coronary angiography, which is less sensitive to detect early stages of CAV than intravascular ultrasound or optical coherence tomography. However, this grading system is the standard method for grading the extent of CAV. Second, since invasive FFR was not performed, we can only hypothesize that the vFFR reflects his invasive counterpart. In this regard, we have to acknowledge that although this software packages have been validated thoroughly in atherosclerotic heart disease, data on vFFR in HTx patients are lacking. Therefore, further validation studies comparing vFFR vs pressure wire-derived vFFR in CAV are warranted. Third, the data presented addresses only pressure losses (i.e., vFFR). Data on the endothelial shear stress that can be calculated with novel software solutions were not analyzed. Finally, this study is limited by its retrospective, observational single center approach and by the small number of patients included in the final analysis.

## 5. Conclusion

Our study suggest that angiography-derived FFR has a diagnostic potential for the detection of allograft vasculopathy in heart transplant recipients. Angiography-derived FFR show an impairment in epicardial conductance in a subgroup of patients without CAV according to the standard ISHLT criteria suggestive for the presence of a diffuse vasculopathy undetectable by conventional coronary angiography. Therefore, we speculate that angiography-derived FFR may be useful in risk stratification after heart transplant.

## Figures and Tables

**Table 1:** Baseline clinical characteristics.

Clinical characteristics	*n* = 65
Age at heart transplant, years, mean ± SD	53.7 ± 10.1
Age at angiography, years, mean ± SD	63.3 ± 12.4
Donor age, years, mean ± SD	35.7 ± 14.4
Donor sex, male, *n* (%)	50 (76.9)
Recipient sex, male, *n* (%)	44 (67.7)
Arterial hypertension, *n* (%)	28 (43.1)
Diabetes mellitus, *n* (%)	25 (38.5)
Hypercholesterolemia, *n* (%)	60 (89.6)
Smoking, *n* (%)	1 (1.5)
History of ischemic cardiomyopathy, *n* (%)	26 (40.0)
History of valvular cardiomyopathy, *n* (%)	5 (7.7)
Other cardiomyopathies, *n* (%)	34 (52.3)
Mycophenolate mofetil, *n* (%)	47 (72.3)
Azathioprine, *n* (%)	2 (3.1)
Calcineurin inhibitors, *n* (%)	65 (100)

SD, standard deviation.

**Table 2:** Functional characteristics by the vFFR analysis.

	*n* = 173
vFFR, median (IQR)	0.88 (0.79, 0.94)
vFFR value ≤0.80, *n* (%)	48 (27.7)
Lesion length (mm), median (IQR)	16.8 (7.60, 30.7)
MLD (mm), mean ± SD	1.70 ± 0.69
Diameter stenosis (%), median (IQR)	35.1 ± 14.0
Reference vessel diameter (mm), mean ± SD	2.60 ± 0.84

vFFR, vessel fractional flow reserve derived from angiography; IQR, interquartile range; MLD, minimal lumen diameter; and SD, standard deviation.

**Table 3:** Functional characteristics according to the CAV grade.

	CAV 0 (*n* = 23)	CAV 1 (*n* = 12)	CAV 2 (*n* = 4)	CAV 3 (*n* = 8)	*p* value
vFFR, median (IQR)	0.84 (0.80, 0.88)	0.76 (0.70, 0.89)	0.72 (0.53, 0.80)	0.54 (0.47, 0.78)	0.009
Lesion length (mm), median (IQR)	26.0 (14.7, 45.5)	22.2 (17.8, 30.0)	31.2 (29.0, 70.7)	36.2 (18.8, 48.4)	0.51
MLD (mm), median (IQR)	1.53 (1.26, 1.72)	1.65 (0.89, 1.73)	0.92 (0.88, 1.62)	0.80 (0.59, 1.38)	0.08
Diameter stenosis (%), median (IQR)	31.0 (25.0, 42.0)	36.0 (31.0, 43.0)	57.0 (56.5, 60.5)	64.0 (59.5, 72.5)	<0.001
Reference diameter (mm), median (IQR)	2.29 (2.03, 2.70)	2.51 (1.54, 2.79)	2.33 (2.23, 3.79)	2.88 (1.67, 3.51)	0.85

CAV, cardiac allograft vasculopathy; vFFR, vessel fractional flow reserve derived from angiography; IQR, interquartile range; and MLD, minimal lumen diameter.

**Table 4:** Agreement between the vFFR value and CAV score.

	vFFR > 0.80	vFFR ≤ 0.80	Total
CAV 0	15	8	23 (48.9)
CAV 1	6	6	12 (25.5)
CAV 2	1	3	4 (8.5)
CAV 3	0	8	8 (17.1)
Total	22 (46.8)	25 (53.2)	47

Values are *n* or *n* (%). vFFR, vessel fractional flow reserve derived from angiography; CAV, cardiac allograft vasculopathy.

## Data Availability

The data used to support the findings of this study are available from the corresponding author upon request.
